# Supplementary material for: Pseudocapacitance-Enhanced Storage Kinetics of 3D Anhydrous Iron (III) Fluoride as a Cathode for Li/Na-Ion Batteries
Source: Nanomaterials (Basel). 2022 Nov 17;12(22):4041. doi: 10.3390/nano12224041 (PMC9692736; doi:10.3390/nano12224041)
Supplement: Supplementary file 1 [file nanomaterials-12-04041-s001.zip › nanomaterials-1989227-SI.pdf]

Supporting Information

Article

Pseudocapacitance-Enhanced Storage Kinetics of 3D Anhydrous Iron (III) Fluoride as a Cathode for Li/Na-Ion Batteries

Tao Zhang †, Yan Liu †, Guihuan Chen *, Hengjun Liu, Yuanyuan Han, Shuhao Zhai, Leqing Zhang, Yuanyuan Pan , Qinghao Li and Qiang Li *

College of Physics, Weihai Innovation Research Institute, College of Materials, Qingdao University, Qingdao 266071, China

* Correspondence: chenguihuan@qdu.edu.cn (G.C.); liqiang@qdu.edu.cn (Q.L.)

† These authors contributed equally to this work.

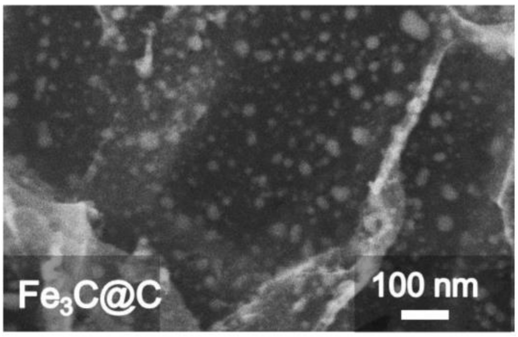

Figure S1. FESEM image of the Fe_3C@C at high magnification.

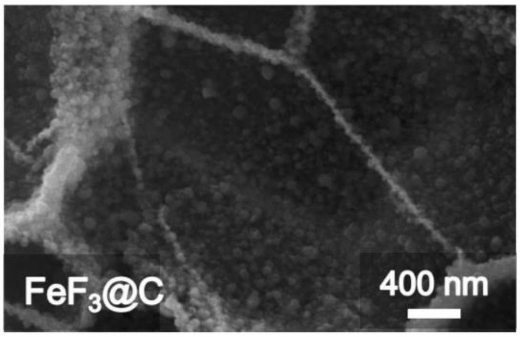

Figure S2. FESEM image of the FeF_3@C at high magnification.

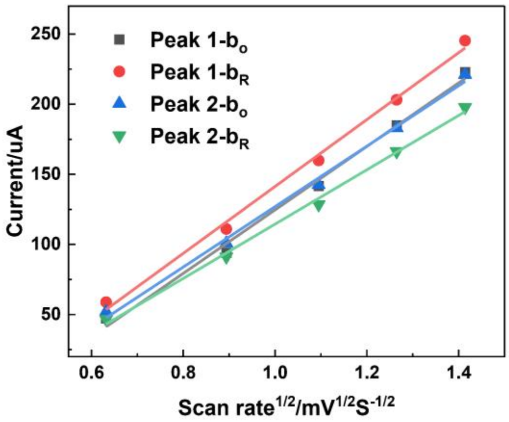

Figure S3. The peak current corresponding to oxidation-reduction is linearly fitted to the square root of the scanning rate in the LIBs.

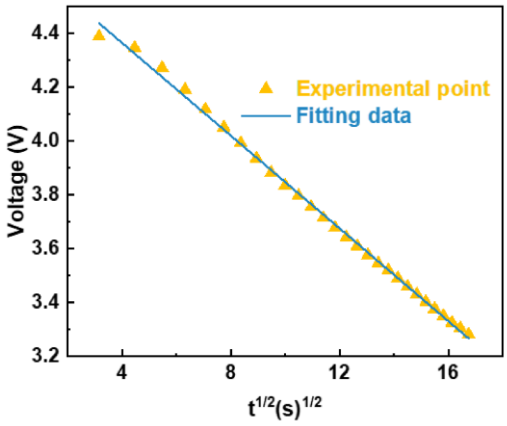

Figure S4. The linear behavior between $t^{1/2}$ and voltage in the LIBs.

Table S1. D_{Li^+} comparison of various fluoride electrodes.

Method	Materials	Magnitude of D_{Li^+} (cm^2s^{-1})	Reference
**GITT**	FeF_3/C	10^{-11}~10^{-9}	[1]
**GITT**	CoF_2/Fe_2O_3	10^{-13}~10^{-12}	[2]
**EIS**	FeF_3/C	10^{-16}~10^{-15}	[3]
**EIS**	FeF_3 • $0.33H_2O$ (nanoparticle)	10^{-12}~10^{-11}	[4]
**EIS**	FeF_3 • $0.33H_2O$ (MOF)	10^{-15}~10^{-14}	[5]

**EIS**	FeF_3/graphitic carbon	10^{-15}~10^{-14}	[6]
**GITT**	FeF_3@C	10^{-12}~10^{-11}	This work

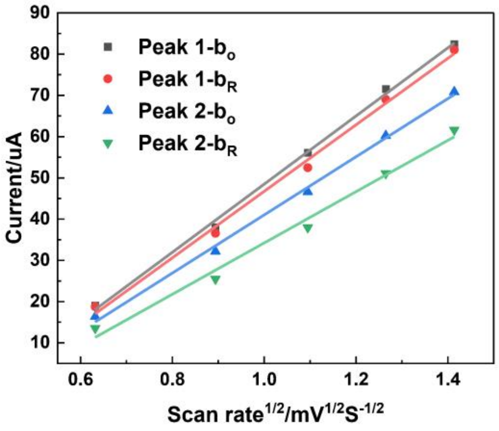

Figure S5. The peak current corresponding to oxidation-reduction is linearly fitted to the square root of the scanning rate in SIBs.

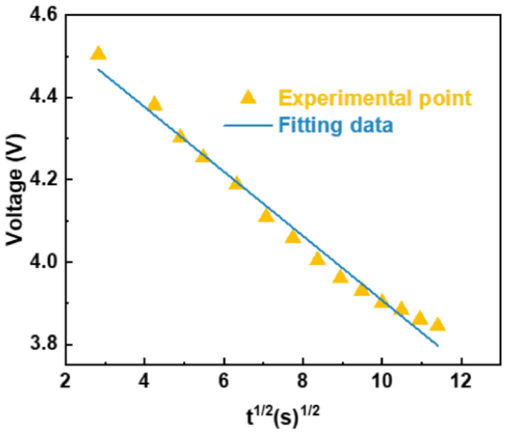

Figure S6. The linear behavior between $t^{1/2}$ and voltage in the SIBs.

Table S2. D_{Na^+} comparison of various fluoride electrodes.

Method	Materials	Magnitude of D_{Na^+} (cm^2s^{-1})	Reference
**GITT**	$FeF_3 \cdot 0.33H_2O$/rGO	10^{-14}~10^{-12}	[7]
**GITT**	FeF_3/graphene	10^{-13}~10^{-11}	[8]

**EIS**	$FeF_3 \cdot 0.33H_2O$@3D-OMCs	10^{-15}~10^{-14}	[9]
**EIS**	FeF_2@NGC	10^{-16}~10^{-14}	[10]
**GITT**	FeF_3@C	10^{-13}~10^{-11}	This work

References

1. Du, K.; Tao, R.; Guo, C.; Li, H.; Liu, X.; Guo, P.; Wang, D.; Liang, J.; Li, J.; Dai, S.; Sun, X.-G. In-situ synthesis of porous metal fluoride@carbon composite via simultaneous etching/fluorination enabled superior Li storage performance. *Nano Energy* **2022**, *103*
2. Cheng, Q.; Chen, Y.; Lin, X.; Liu, J.; Yuan, Z.; Cai, Y. Hybrid Cobalt(II) Fluoride Derived from a Bimetallic Zeolitic Imidazolate Framework as a High-Performance Cathode for Lithium–Ion Batteries. *J. Phys. Chem. C* **2020**, *124*, 8624-8632.
3. Li, J.; Fu, L.; Xu, Z.; Zhu, J.; Yang, W.; Li, D.; Zhou, L. Electrochemical properties of carbon-wrapped FeF3 nanocomposite as cathode material for lithium ion battery. *Electrochim. Acta* **2018**, *281*, 88-98.
4. Lin, J.; Chen, S.; Zhu, L.; Yuan, Z.; Liu, J. Soft-template fabrication of hierarchical nanoparticle iron fluoride as high-capacity cathode materials for Li-ion batteries. *Electrochim. Acta* **2020**, *364*, 137293.
5. Cheng, Q.; Pan, Y.; Chen, Y.; Zeb, A.; Lin, X.; Yuan, Z.; Liu, J. Nanostructured Iron Fluoride Derived from Fe-Based Metal-Organic Framework for Lithium Ion Battery Cathodes. *Inorg. Chem.* **2020**, *59*, 12700-12710.
6. Kim, T.; Jae, W.J.; Kim, H.; Park, M.; Han, J.M.; Kim, J. A cathode material for lithium-ion batteries based on graphitized carbon-wrapped FeF3 nanoparticles prepared by facile polymerization. *J. Mater. Chem. A* **2016**, *4*, 14857-14864.
7. Zhang, C.; An, S.; Li, W.; Xu, H.; Hao, W.; Liu, W.; Li, Z.; Qiu, X. Hierarchical Mesoporous Iron Fluoride and Reduced Graphene Oxide Nanocomposite as Cathode Materials for High-Performance Sodium-Ion Batteries. *ACS Appl. Mater. Interfaces* **2020**, *12*, 17538-17546.
8. Shen, Y.; Wang, X.; Hu, H.; Jiang, M.; Bai, Y.; Yang, X.; Shu, H. Sheet-like structure FeF3/graphene composite as novel cathode material for Na ion batteries. *RSC Adv.* **2015**, *5*, 38277-38282.
9. Zhang, R.; Wang, X.; Wang, X.; Liu, M.; Wei, S.; Wang, Y.; Hu, H. Iron Fluoride Packaged into 3D Order Mesoporous Carbons as High-Performance Sodium-Ion Battery Cathode Material. *J. Electrochem. Soc.* **2018**, *165*, A89-A96.
10. Yanuar Maulana, A.; Song, J.; Futalan, C.M.; Kim, J. Improved reversibility of phase transformations using electron-rich graphitic carbon matrix in FeF2 cathode for sodium-ion batteries. *Chem. Eng. J.* **2022**, *434*.
